# Fibroblast growth factor 21 resistance is associated with body shape in patients with type 2 diabetes complicating hypertension

**DOI:** 10.3389/fcvm.2023.1168047

**Published:** 2023-06-22

**Authors:** Jing Gan, Zikun Duan, Lu Tang, Zhen Liu, Huiying Tian, Maolan Wu, Yanxue Bi, Xingchao Pan, Wenjun Wang, Xiaotang Gao, Ningrui Wang, Zhuofeng Lin, Hong Yang

**Affiliations:** ^1^ Department of Cardiology, the 1st affiliated Hospital of Wenzhou Medical Unversity, Wenzhou, China; ^2^ School of Pharmaceutical College, Wenzhou Medical University, Wenzhou, China; ^3^ Laboratory Animal Center of Wenzhou Medical University, Wenzhou, China; ^4^ Department of Endocrinology, the 3rd Affiliated Hospital of Wenzhou Medical University, Ruian People’s Hospital, Wenzhou, China

**Keywords:** FGF21 resistance, body shape, waistline, type 2 diabetes mellitus, hypertension

## Abstract

**Objectives:**

Obesity, especially abdominal obesity, increases the prevalence of metabolic and cardiovascular disease (CVD). Fibroblast growth factor 21 (FGF21) has been identified as a critical regulator playing a therapeutic role in diabetes and its complications. This study aims to evaluate the relationship between serum FGF21 levels and body shape parameters in patients with hypertension (HP) and type 2 diabetes mellitus (T2DM).

**Methods:**

Serum FGF21 levels were determined in 1,003 subjects, including 745 patients with T2DM, and 258 individuals were selected as a healthy control in this cross-sectional study.

**Results:**

Serum FGF21 levels were significantly higher in T2DM patients with HP than those without [534.9 (322.6–722.2) vs. 220.65 (142.8–347.55) pg/ml, *p* < 0.001], and levels in both of these two groups were significantly increased compared with that of healthy control [123.92 (67.23–219.32) pg/ml, all *p* < 0.001]. These differences were also observed in body shape parameters, including weight, waistline, body mass index (BMI), body shape index (ABSI), and the percentage of abdominal obesity. Serum FGF21 levels in T2DM patients were positively correlated with body shape parameters, including weight, waistline, neck circumference, BMI, ABSI, percent of abdominal obesity, and triglyceride, while negatively with estimated glomerular filtration rate (all *p* < 0.01). The significance remained stable when adjusted for age and T2DM duration. In addition, both serum FGF21 concentrations and waistline were independently associated with HP in T2DM patients after the adjustment for risk factors (all *p* < 0.05). ROC analysis for FGF21 levels of 745 patients with T2DM identified 411.33 pg/ml as an optimal cut-off point to predict HP, with a sensitivity and specificity of 66.0% and 84.9%, respectively.

**Conclusions:**

FGF21 resistance occurs in patients of HP in T2DM, and positively correlates with body shape parameters (especially waistline and BMI). High levels of FGF21 may be a compensatory reaction to offset HP.

## 1. Introduction

Metabolic syndrome is a combination of multiple metabolic disorders, including obesity, insulin resistance, hyperinsulinemia, abnormal lipid metabolism, abnormal glucose metabolism and hypertension (HP) (1). These metabolic disorders are the pathological basis of heart, cerebrovascular diseases, and diabetes. It is generally recognized that the common cause of these disorders is obesity, especially abdominal obesity, which results in the body's resistance to insulin, thus reducing the utilization of peripheral glucose and increase the utilization of fatty acids, leading to type 2 diabetes mellitus (T2DM) (2). In addition, it should be noted that similar manifestations of metabolic syndrome could also be observed in individuals with abdominal obesity but not overweight in total body weight (3). Obesity-induced insulin resistance and associated hyperinsulinemia, hyperglycemia, and adipokine may also cause vascular endothelial dysfunction, dyslipidemia, HP, and vascular inflammation, which contribute to the development of atherosclerotic cardiovascular disease (CVD) (4, 5). Controlling obesity is a standard preventive and therapeutic measure for metabolic disorders (6).

Recently, fibroblast growth factor 21(FGF21) has been considered as a novel metabolic risk factor, which is closely associated with a variety of metabolic and CVD (7, 8). Circulating FGF21 is mainly derived from the liver and acts by binding to its receptor FGFR1 and coreceptor β-klotho in regulating energy balance and glucose and lipid homeostasis (9). Surprisingly, however, individuals with metabolic disorders such as obesity, T2DM and fatty liver have high levels of FGF21 and respond poorly to exogenous FGF21, this phenomenon was defined as FGF21 resistance. FGF21 resistance is a state of the body's weakened response to FGF21, which is manifested by reduced biological effects of target tissues and increased compensatory synthesis of FGF21 in the body (10, 11). This may be due to decreased expression or sensitivity of the FGF21 receptor and β-klotho protein (12). Long-term lifestyle interventions such as diet, exercise, weight control, etc. may improve FGF21 resistance (10). These changes in daily habits are equally important in combination of antihypertensive drugs for controlling blood pressure. Therefore, it is of great importance to motivate people to keep their body in good shape.

In non-metabolic CVDs, FGF21 plays an essential role in the regulation of blood pressure. In mechanism, our previous study found that FGF21 participated in the regulation of HP by improving the function of endothelial cells, promoting the release of vasodilatory factors, inhibiting vascular remodeling, and antagonizing angiotensin II (Ang II). By using FGF21 gene knockout mice and overexpressed mice, we confirmed the effect of FGF21 on lowering blood pressure and protecting the cardiovascular system. FGF21 protects against Ang II-induced HP and vascular impairment by fine-tuning the multi-organ crosstalk between liver, adipose tissue, kidney, and blood vessels through the activation of the ACE2/Ang-(1–7) axis. It is a medium for multiple organs and systems to resist pathological changes jointly (13). In addition, the antihypertensive treatment prepared by FGF21 as the active substance has a significant improvement effect on HP symptoms caused by Ang II (14). Basic research and clinical application of FGF21 in blood pressure regulation has made preliminary progress in recent years (15). Compared with traditional antihypertensive drugs such as *β*-blockers, angiotensin-converting enzyme inhibitors, diuretics, etc., FGF21 not only improves blood pressure but also has the function of protecting the cardiovascular system and alleviating vascular injury, showing the potential of FGF21 in the development of drugs for dealing with HP. On the one hand, FGF21 promotes the development of HP, possibly through fluid retention. FGF21 is a critical mediator in PPAR*γ* signal transduction. Mice with FGF21 gene knockout showed PPAR*γ* signal defects and resistance to significant side effects (such as weight gain and edema) of rosiglitazone (PPAR*γ* agonist) (16). On the other hand, FGF21 is also a hormone that acts on the central nervous system. It can enter the human blood circulation, cross the blood-brain barrier, and its receptor FGFR and cofactor *β*-clotho are distributed in key locations of pressure emission pathways and blood pressure regulation sites. Therefore, FGF21 may be involved in the regulation of central blood pressure by activating related signaling proteins in the central nervous system (17). However, more and more studies have reported that the elevated human serum FGF21 level was independently associated with HP, obesity, T2DM and fatty liver diseases, which led us to hypothesize that HP in fact may affect the raise of serum FGF21 level, especially in the state of diabetes.

For the superior efficacy of FGF21in handling metabolic diseases, including HP, thus improving FGF21 resistance is a new idea for the treatment of obesity and related metabolic disease. In the general population, FGF21 level has been confirmed to be closely associated with metabolic syndrome, including obesity, non-alcoholic fatty liver disease, HP, and diabetes (18). Complex metabolic diseases such as obesity, HP, diabetes, etc. are isolated and interrelated. However, the relationship between FGF21, body shape, and blood pressure in patients with diabetes remains unknown. In this regard, the purpose of this study is to systematically explore the correlation between serum FGF21 levels of HP in patients with T2DM and to find out the risk factors of diabetes complicated with HP to provide new methods for the etiology, early screening, diagnosis, treatment, and prevention of chronic complications of diabetes.

## 2. Materials and methods

### 2.1. Participants

A total of 745 patients diagnosed with T2DM participated in this cross-sectional study recruited from July 1, 2019–January 28, 2023 from the Diabetes Center of Ruian People's Hospital. T2DM was diagnosed according to the criteria of DM proposed by the World Health Organization in 1999: Symptoms of diabetes, a blood glucose level of 11.1 mmol/L at any time, a fasting blood glucose (FPG) level of 7.0 mmol/L or a 2-hour blood glucose level of 11.1 mmol/L (19). HP was defined with systolic blood pressure (SBP) ≥ 140 mmHg, diastolic BP (DBP) ≥ 90 mmHg, or a history of HP, or use of antihypertensive drugs. Body mass index (BMI) was defined as weight over height^2^ (kg/m^2^), a body shape index (ABSI) was calculated as waistline/(BMI^2/3^height^1/2^) (m^11/6^kg^−2/3^), overall obesity was defined by BMI ≥ 28 kg/m^2^, and abdominal obesity was defined by waistline ≥ 90 cm for men and ≥85 cm for women. A complete history and thorough physical examination were obtained in each case. BP was measured using a mercury sphygmomanometer after a 10 min rest. Waistline to hipline ratio (WHR), headline, and neckline was also measured at baseline. In addition, routine laboratory tests were done. This study was performed with the approval of the Human Ethics Committee of Ruian People's Hospital, and written informed consent was obtained from each participant.

### 2.2. Clinical data collection and biochemical assessment

Additional laboratory data and detailed history of physician-diagnosed disease, drinking, and smoking histories were obtained and evaluated through questionnaires. Blood was drawn following an 8–12 h period of fasting for determination of FPG, hemoglobin A1c (HbA1c), triglyceride (TG), total cholesterol (TC), high-density lipoprotein cholesterol (HDL-c), and low-density lipoprotein cholesterol (LDL-c). The chronic kidney disease epidemiology collaboration formula was used to calculate the estimated glomerular filtration rate (eGFR). Serum samples obtained from all participants were stored in −80°C preparing for further detection. Human serum FGF21 levels were quantitatively determined *in vitro* by a standard enzyme-linked immunosorbent assay kit (Antibody and Immunoassay Service, HKU, HK). Serum FGF21 levels and other baseline characteristics were compared between T2DM patients with and without HP. Univariable and multivariable logistic regression analysis was used to identify factors most closely associated with HP. Furthermore, correlation analysis was performed to examine the determinant factors of circulating FGF21 levels in patients with T2DM.

### 2.3. Statistical analysis

All statistical analyses were performed using Statistical Package for Social Science Version 26.0 (IBM Corporation, NY, USA). Continuous data were expressed as mean ± standard deviation or median with interquartile range. Categorical data are expressed as percentages. Independent-sample *t* test, one-way ANOVA followed by Tukey's HSD test or the nonparametric Kruskal-Wallis test followed by Dunn's test, and *χ*^2^ test was used to determine differences between groups. Variables with statistical significance between groups and variables which were biologically likely to be related to HP were all included to determine independent factors of HP in T2DM, using univariable and multivariable logistic regression analysis. In addition, pearson correlation and partial correlation analysis adjusted for age and T2DM duration were applied to identify the association of FGF21 and body shape parameters or biochemical parameters. The predictive value of FGF21 against HP was determined with ROC curves. Two-sided *p* values < 0.05 were considered statistically significant.

## 3. Results

### 3.1. Serum FGF21 levels are significantly increased in T2DM patients with or without HP compared to healthy controls

The physical and biochemical characteristics of T2DM patients and healthy controls including FGF21 levels, body shape parameters, and biochemical parameters likely to be related to HP risks are summarized in Table 1. Serum FGF21 levels was increased in T2DM patients compared with those in healthy [220.65 (142.8–347.55) vs. 123.92 (67.23–219.32) pg/ml, all *p* < 0.001]. Moreover, T2DM patients with HP expressed at the FGF21level of more than twice as high as those without [534.9 (322.6–722.2) vs*.* 220.65 (142.8–347.55) pg/ml, *p* < 0.001]. There was no statistically significant difference between patients of diabetes with or without HP on height, significant differences were only found when compared with healthy, however, there was a gradual increase in weight in these three groups (59.10 ± 10.14 vs.67.90 ± 11.16 vs. 71.51 ± 11.76 kg, all *p* < 0.001), so was BMI (22.28 ± 2.92 vs. 24.86 ± 3.069 vs. 26.53 ± 3.40 kg/m^2^, all *p* < 0.001). WHR was equal to waistline/hipline. The mean hipline of the patients of diabetes with or without HP was nearly equal (74.14 ± 14.07 vs. 74.17 ± 14.60 cm, *p* = 0.974); interestingly, however, both waistline (92.83 ± 8.78 vs. 87.70 ± 8.38 cm, *p* < 0.001) and WHR (1.30 ± 0.28 vs. 1.22 ± 0.25, *p* < 0.001) were increased in diabetes with HP as compared to those without. Similarly, elevated ABSI (0.082 ± 0.004 vs. 0.08 ± 0.003 m^11/6^kg^−2/3^) and neckline (38.29 ± 3.17 vs. 37.40 ± 3.79 cm) were found in these T2DM individuals with HP compared with those without (all *p* < 0.001). In addition, the prevalence of abdominal obesity was significantly higher in diabetes with HP (27.6 vs. 15.8%, *p* < 0.001) than that of diabetes alone. No significant difference was found in headline between the two groups (56.47 ± 2.64 vs. 56.72 ± 2.25 cm, *p* = 0.173). Although there was a significant difference in anti-diabetic drugs use (Table 1), no correlation was found between the anti-diabetic treatments and serum FGF21 levels (Table 2).

**Table 1:** Baseline characteristics of the study participants with HP.

Variables	Healthy (*n* = 258)	T2DM_No_HP (*n* = 404)	T2DM_HP (*n* = 341)	*p* T2DM_HP vs. Healthy	*p* T2DM_No_HP vs. Healthy	*p* T2DM_HP vs. T2DM_No_HP
FGF21, pg/ml	123.92 (67.23–219.32)	220.65 (142.8–347.55)	534.9 (322.6–722.2)	<0.001	<0.001	**<0** **.** **001**
**Body shape variables**						
Height, cm	162.57 ± 7.62	164.98 ± 8.02	163.94 ± 7.55	0.028	<0.001	0.069
Weight, kg	59.10 ± 10.14	67.90 ± 11.16	71.51 ± 11.76	<0.001	<0.001	**<0** **.** **001**
Waistline, cm	/	87.70 ± 8.38	92.83 ± 8.78	/	/	**<0** **.** **001**
Hipline, cm	/	74.14 ± 14.07	74.17 ± 14.60	/	/	0.974
Headline, cm	/	56.47 ± 2.64	56.72 ± 2.25	/	/	0.173
Neckline, cm	/	37.40 ± 3.79	38.29 ± 3.17	/	/	**<0** **.** **001**
BMI, kg/m^2^	22.28 ± 2.92	24.86 ± 3.069	26.53 ± 3.40	<0.001	<0.001	**<0** **.** **001**
ABSI, m^11/6^kg^−2/3^	/	0.08 ± 0.003	0.082 ± 0.004	/	/	**<0** **.** **001**
WHR	/	1.22 ± 0.25	1.30 ± 0.28	/	/	**<0** **.** **001**
Abdominal obesity, %	/	15.8	27.6	/	/	**<0** **.** **001**
**General and biochemical variables**						
Male, %	41.9	74.8	73.9	<0.001	<0.001	0.801
Current smoking, %	16.6	37.4	35.8	<0.001	<0.001	0.703
Current drinking, %	19.5	33.2	26.1	0.103	0.001	**0** **.** **037**
Age, years	41.36 ± 12.25	49.03 ± 11.03	56.82 ± 7.99	<0.001	<0.001	**<0** **.** **001**
T2DM duration, months		59.72 ± 73.86	97.06 ± 88.03			**<0** **.** **001**
FPG, mmol/L	4.82 (4.51–5.11)	7.7 (6.3–9.65)	7.3 (6.1–9.2)	<0.001	<0.001	0.093
HbA1c, %	5.4 (5.17–5.6)	8.9 (7.4–11.1)	8.0 (7.1–9.6)	<0.001	<0.001	**<0** **.** **001**
SBP, mmHg	117.84 ± 13.04	126.81 ± 17.24	143.44 ± 19.34	<0.001	<0.001	**<0** **.** **001**
DBP, mmHg	70.49 ± 9.09	73.86 ± 10.44	79.32 ± 11.10	<0.001	<0.001	**<0** **.** **001**
TG, mmol/L	1.13 (0.81–1.57)	1.5 (1.0–2.3)	1.8 (1.2–2.4)	<0.001	<0.001	**0** **.** **002**
TC, mmol/L	4.95 (4.32–5.51)	4.7 (4.0–5.5)	4.6 (3.9–5.3)	0.074	0.386	0.311
HDL-c, mmol/L	1.36 (1.14–1.54)	1.0 (0.9–1.2)	1.0 (0.9–1.2)	<0.001	<0.001	0.762
LDL-c, mmol/L	3.11 (2.56–3.72)	2.9 (2.3–3.5)	3.7 (2.1–3.4)	0.009	0.264	0.080
eGFR, ml/min/1.73m^2^	118.49 (109.18–128.38)	109.7 (100.8–117.15)	101.1 (90.9–107.6)	<0.001	<0.001	**<0** **.** **001**
**Anti-diabetic drugs**						
No, %	/	33.4	22.6	/	/	**0** **.** **001**
Insulin, %	/	8.2	7.0	/	/	**0** **.** **583**
OHA, %	/	40.8	39.3	/	/	**0** **.** **708**
Insulin + OHA, %	/	18.6	30.8	/	/	**<0** **.** **001**

Data are presented as mean ± SD, or median (interquartile range); FGF21, fibroblast growth factor 21; BMI, body mass index; ABSI, a body shape index; WHR, waistline to hipline ratio; FPG, fasting plasma glucose; HbA1c, haemoglobin A1c; SBP, systolic blood pressure; DBP, diastolic blood pressure; TG, triglyceride; TC, total cholesterol; HDL-c, high-density lipoprotein cholesterol; LDL-c, low-density lipoprotein cholesterol; eGFR, estimated glomerular filtration rate; No, no hypoglycemic agents use; OHA, oral hypoglycemic agent.

The use of bold emphasis is to mark the statistically significant indicators.

**Table 2:** Correlation of LnFGF21 with body shape and other risk factors of HP in patients with T2DM.

	Serum LnFGF21		Serum LnFGF21 (age-adjusted)		Serum LnFGF21 (age-and T2DM duration-adjusted)	
Variables	*r*	*p*	*r*	*p*	*r*	*p*
Weight	0.109	**0** **.** **003**	0.152	**<0** **.** **001**	0.152	**<0** **.** **001**
Waistline	0.202	**<0** **.** **001**	0.200	**<0** **.** **001**	0.200	**<0** **.** **001**
Neckline	0.061	0.097	0.085	**0**.**021**	0.086	**0**.**021**
BMI	0.204	**<0** **.** **001**	0.221	**<0** **.** **001**	0.221	**<0** **.** **001**
ABSI	0.104	**0** **.** **005**	0.052	0.159	0.052	0.158
WHR	0.009	0.816	0.001	0.986	0.002	0.958
Abdominal obesity	0.115	**<0** **.** **001**	0.131	**<0** **.** **001**	0.131	**<0** **.** **001**
Current drinking	−0.048	0.107	−0.063	0.089	−0.063	0.090
Age	0.145	**<0** **.** **001**				
T2DM duration	0.052	0.158	−0.003	0.933		
HbA1c*	−0.028	0.444	0.007	0.845	0.007	0.848
TG*	0.174	**<0** **.** **001**	0.210	**<0** **.** **001**	0.210	**<0** **.** **001**
eGFR*	−0.163	**<0** **.** **001**	−0.112	**0**.**002**	−0.113	**0**.**002**
HP	0.408	**<0** **.** **001**	0.405	**<0** **.** **001**	0.410	**<0** **.** **001**
No anti-diabetic use	−0.028	**0** **.** **346**	0.011	**0**.**760**	0.011	**0**.**764**
Insulin + OHA	0.018	**0** **.** **552**	−0.039	**0**.**287**	−0.040	**0**.**279**

* Log transformed before analysis.

The use of bold emphasis is to mark the statistically significant indicators.

In sum, FGF21 is increased, and a worse of body shape parameters, including elevated weight, waistline, neckline, BMI, ABSI, and WHR, was found in these T2DM individuals with HP compared with those without this complication (all *p *< 0.05). These data suggest FGF21 resistance occurs in diabetes, especially those with HP.

### 3.2. Serum FGF21 levels are closely correlated with a cluster of body shape parameters and HP in patients with T2DM

The prevalence of abdominal obesity is high, and an increased serum level of FGF21 was observed in HP individuals with diabetes (Table 1). Is there any relationship between serum FGF21 levels and body shape in these T2DM individuals? We next investigated the relationship between serum FGF21 levels and a cluster of body shape parameters. Correlation analysis showed a significant positive association of serum FGF21 levels with body shape parameters, including weight, waistline, BMI, ABSI, abdominal obesity, and TG, respectively (all *p *< 0.05). However, serum FGF21 was negatively correlated with eGFR (*p* < 0.001). After the adjustment for age, the significance remained stable, with a bit of change in neckline (*p *< 0.05) and ABSI (*p *> 0.05). Furthermore, the correlation of serum FGF21 with these parameters, except for ABSI remained significant even after the adjustment for age and T2DM duration (Table 2). To clarify whether HP is correlated with FGF21, we also analyzed the relationship of serum FGF21 with HP by partial correlation analysis. As shown in Table 2, HP was strikingly positively correlated with serum FGF21 levels when adjusted by age and T2DM duration (*r* = 0.410, *p* < 0.001). These data suggest that serum FGF21 levels are closely related to body shape and HP in patients with T2DM.

### 3.3. Serum FGF21 levels and waistline are independently associated with HP in T2DM patients

To investigate whether FGF21 is related to the morbidity of HP in patients with T2DM, we first explored the risk factors of HP in these T2DM subjects. As shown in Table 1, an increased manner of serum FGF21 levels was observed in these T2DM subjects with HP compared with those without. Consistent with the change of FGF21, a higher prevalence of abdominal obesity was observed in these individuals. In univariable logistic regression analyses (Table 3), body weight, waistline, neckline, BMI, ABSI, and WHR, but not standing height or hipline, predicted the occurrence of HP in these T2DM subjects, suggesting that body weight, waistline, neckline, BMI, ABSI, and WHR screening in diabetic patients could be helpful in detecting high-risk individuals of cardiovascular complications such as HP as early as possible. In addition, current drinking, age, T2DM duration, HbA1c, TG, and eGFR were also found to be independent risk factors for the established HP. In stepwise multiple regression analysis (Table 4), the predictors of HP were FGF21, weight, and waistline in the model adjusted by body shape parameters. FGF21, age, T2DM duration, HbA1c, TG, and eGFR are risk factors for HP in the model adjusted by general and biochemical parameters. Interestingly, the association of serum FGF21, waistline, age, HbA1c, and eGFR with HP remained significant in model 3, which was adjusted by parameters with *p* < 0.05 in models 1–2.

**Table 3:** Factors associated with HP in patients with T2DM in univariable logistic regression analyses (*n* = 745).

Variables	*OR* (95% CI)	*p* value
FGF21*	4.607 (3.542–5.993)	**<0** **.** **001**
**Body shape variables**		
Height	0.983 (0.965–1.001)	0.070
Weight	1.028 (1.015–1.042)	**<0** **.** **001**
Waistline	1.074 (1.054–1.094)	**<0** **.** **001**
Hipline	1.000 (0.990–1.010)	0.974
Neckline	1.080 (1.032–1.129)	**0** **.** **001**
BMI	1.178 (1.122–1.237)	**<0** **.** **001**
ABSI	8.223E + 48 (1.756E + 30–3.851E + 67)	**<0** **.** **001**
WHR	3.010 (1.711–5.296)	**<0** **.** **001**
Abdominal obesity	2.022 (1.414–2.890)	**<0** **.** **001**
**General and biochemical variables**		
Current drinking	0.712 (0.518–0.978)	**0** **.** **036**
Age	1.088 (1.069–1.108)	**<0** **.** **001**
T2DM duration	1.006 (1.004–1.008)	**<0** **.** **001**
HbA1c*	0.178 (0.093–0.339)	**<0** **.** **001**
TG*	1.350 (1.062–1.717)	**0** **.** **014**
eGFR*	0.016 (0.005–0.049)	**<0** **.** **001**

* Log transformed before analysis.

The use of bold emphasis is to mark the statistically significant indicators.

**Table 4:** Factors associated with HP in patients with T2DM in stepwise multivariable logistic regression analysis (*n* = 745).

Variables	*OR* (95% CI)	*p* value
**Model 1**		
FGF21*	4.200 (3.202–5.509)	**<0** **.** **001**
Weight	0.958 (0.932–0.984)	**0** **.** **002**
Waistline	1.110 (1.069–1.152)	**<0** **.** **001**
**Model 2**		
FGF21*	4.636 (3.474–6.188)	**<0** **.** **001**
Age	1.069 (1.044–1.095)	**<0** **.** **001**
T2DM duration	1.003 (1.001–1.005)	**0** **.** **012**
HbA1c*	0.189 (0.081–0.437)	**<0** **.** **001**
TG*	1.573 (1.142–2.168)	**0** **.** **006**
eGFR*	0.243 (0.066–0.892)	**0** **.** **033**
**Model 3**		
FGF21*	4.422 (3.297–5.93)	**<0** **.** **001**
Waistline	1.048 (1.004–1.093)	**0** **.** **032**
Age	1.089 (1.06–1.119)	**<0** **.** **001**
HbA1c*	0.212 (0.088–0.507)	**<0** **.** **001**
eGFR*	0.224 (0.059–0.85)	**0** **.** **028**

* Log transformed before analysis. Model 1, adjusted by body shape factors including body height, weight, waistline, hipline, neckline, BMI, ABSI, WHR, and abdominal obesity; Model 2, adjusted by general and biochemical variables including current drinking, age, T2DM duration, HbA1c, TG, and eGFR; Model 3, adjusted by variables with *p* < 0.05 in models 1–2.

The use of bold emphasis is to mark the statistically significant indicators.

**Figure 1:**
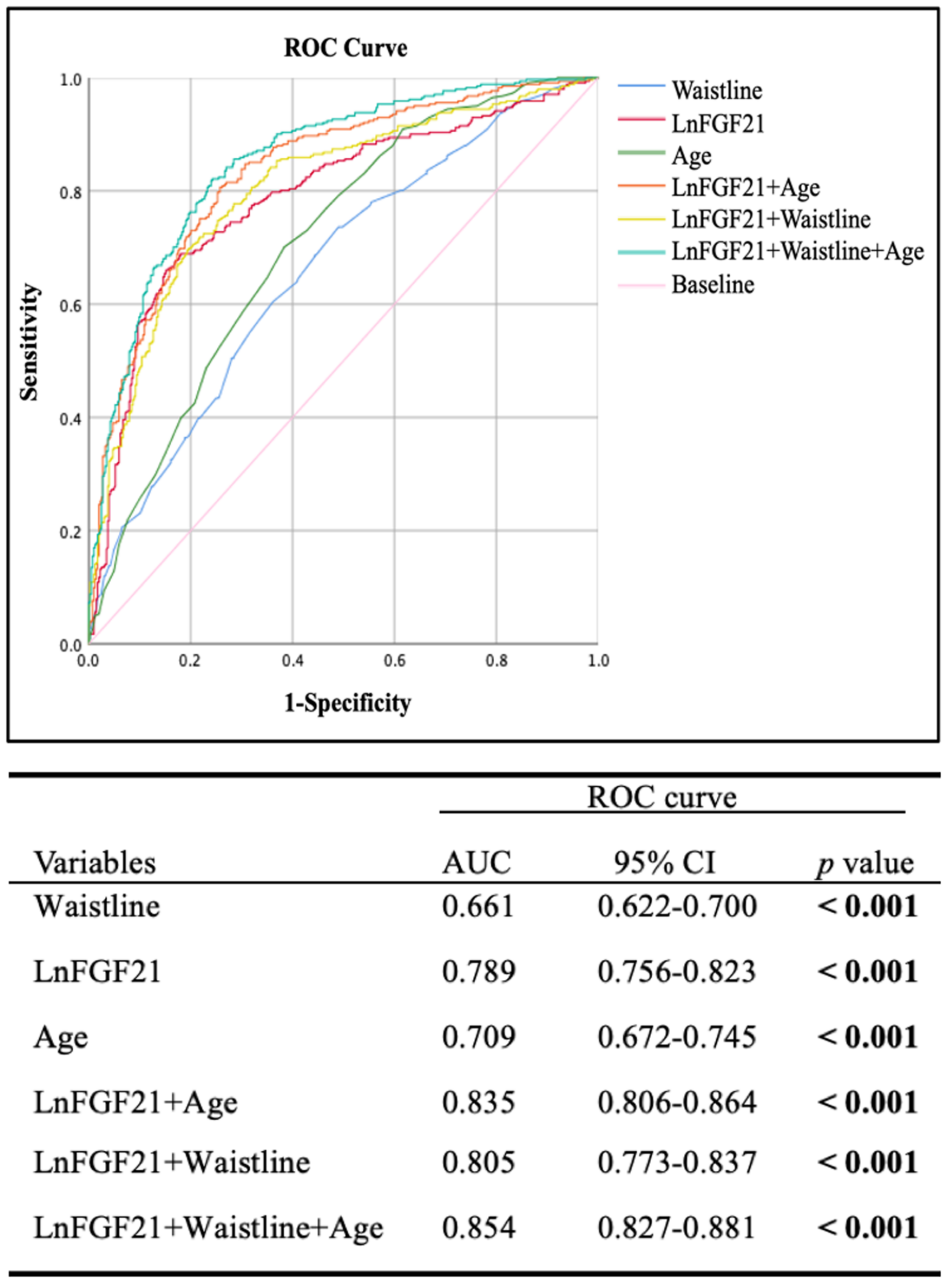
Curve analysis of serum FGF21, waistline, and age for predicting the HP of 745 patients with T2DM. Upper panel: ROC curve; Down panel: the area under ROC (AUC) curve.

In addition, Receiver operating characteristic (ROC) curve analysis and 95% confidence interval (CI) were applied to judge the value of FGF21 on HP high-risk score. In this study, the area under the curve (AUC) was found to be statistically significant (AUC: 0.789, 95% CI: 0.756–0.823, *p* < 0.001) in the total sample (Figure 1). Furthermore, a combined analysis of FGF21, waistline, and age got a more considerable AUC value compared with these three parameters alone (AUC: 0.854, 95% CI: 0.827–0.881, *p* < 0.001), moreover, the sensitivity and specificity of combined detection of FGF21, waistline and age were significantly higher than those of single detection. As an optimal cutoff point, high risk LnFGF21 value of 6.02 pg/ml (FGF21 value of 411.33 pg/ml) was determined with a 66.0% sensitivity and 84.9% specificity. Taken together, serum FGF21 levels (411.33 pg/ml or more), waistline (87.75 cm or more), and age (53.5 year or more) may be good predictors for the incidence of HP in patients with T2DM.

## 4. Discussion

Patients with diabetes are most likely to have a high risk of CVD, which is the leading cause of death among patients with T2DM. HP is a cardiovascular syndrome with elevated systemic arterial pressure as the primary clinical manifestation. Our previous animal research demonstrated that FGF21 plays an essential role in regulating blood pressure (13). However, such a conclusion, even if well founded, is unsatisfactory for due to the few human studies for metabolic cardiovascular diseases evaluating the relationship between FGF21 and HP have been reported. Current studies on FGF21 mainly focus on glucose and lipid metabolism, obesity and diabetes. FGF21 levels in circulation (20), cord blood (21), and placenta (22) were elevated in pregnant women with diabetes mellitus, and positively correlated with human BMI. Surprisingly, however, these findings contradict the positive efficacy of FGF21 in rodents. Therefore, some researchers have questioned whether FGF21 resistance exists in patients with obesity and other related metabolic diseases (23). The expected beneficial effects of endogenous FGF21 are not present in obesity, which is a great challenge for clinical drug development based on FGF21. Given the close relationship between obesity, diabetes and other metabolic diseases and HP, scholars have begun to pay attention to the relationship between FGF21 level and HP in recent years, and studies have shown that circulating FGF21 level is significantly correlated with increased blood pressure (24). These data suggest that complicated metabolic cardiovascular problems may be an FGF21-resistant state.

Nevertheless, the causal relationship and potential effects between HP and FGF21 need to be better clarified. HP may be related to the improvement of insulin resistance and the influence of FGF21 on the afferent pathway of stress reflex. In fructose-induced hypertensive SD rats, FGF21 could improve the absorption and utilization of glucose, improve insulin resistance, increase the activity of NO synthase, stimulate endothelial cells to release strong vasodilator NO, improve endothelial function, and ultimately significantly reduce the SBP of SD rats (25). Our previous studies showed that the inhibition of FGF21 can promote Ang II production, leading to the occurrence of HP. Treatment with recombinant FGF21 can convert Ang II into Ang (1–7), expand the artery to reduce the peripheral resistance of blood vessels, reverse vascular damage, and lower high blood pressure (13). FGF21 can also prevent myocardial fibrosis caused by HP. During HP, both systemic and cardiac FGF21 will be induced and act on the heart to avoid hypertensive heart disease (26). HP is also a chronic inflammatory disease, which can cause the accumulation of pro-inflammatory cells including macrophages and T lymphocytes, which play a crucial role in the pathogenesis of HP (27). Depletion of FGF21 resulted in a significant increase in blood pressure, which led to increased infiltration of monocytes, macrophages and T cells, and aggravated aortic inflammation in Ang II-treated wild-type mice. These negative effects were significantly amplified in FGF21 knockout mice but were entirely reversed by infection with aav-FGF21, showing that FGF21 can inhibit HP and ameliorate HP-mediated vascular damage (28). These above results suggest that FGF21 can regulate blood pressure by regulating renin-angiotensin system, stress reflex afferent pathway, and inhibiting inflammatory response. With further research, FGF21 is expected to become a new biomarker to predict the occurrence of HP, and FGF21 or its agonists may become a new target for the treatment of HP.

It was firstly reported that the elevated serum FGF21 level was independently associated with HP in American community residents according to a prospective open cohort study (20), with the same results in Chinese (29, 30) and Japanese populations (31). In this study, we first evaluated the changes of circulating FGF21 in subjects with and without HP in T2DM patients, and we provided first clinical evidence showing that serum FGF21 levels were significantly increased in patients with T2DM than in healthy controls, and much higher FGF21 levels were observed in patients combined with HP. Serum FGF21 concentrations in these patients with T2DM were independently associated with HP in univariate and multivariate binary logistic regression analyses, similar results to other studies (29–31). There is a significant correlation between FGF21 and HP. However, the mechanisms of FGF21 involved in the development and progression of metabolic syndrome especially in HP plus T2DM as yet unexplored. One of the potential role of phosphatases or other molecules that may impair ERK pathway signaling on FGF21 remains to be determined (23). Further research is needed to investigate the mechanism of FGF21 on the development and progression of metabolic disorders in these T2DM individuals with HP.

Previous studies have shown that body shape is closely related to HP (32), and serum FGF21 level is higher in obese people (33). Similarly, in the current study, the body shape parameters, including body weight, waistline, neck circumference, BMI, ABSI, and WHR of T2DM patients with HP, were significantly higher than those without. The proportion of abdominal obesity in HP was also considerably increased, this may be related to the exacerbation of metabolic syndrome or diabetic chronic complications in these patients with T2DM. As expected, serum FGF21 levels were significantly associated with weight, waistline, BMI, ABSI, and abdominal obesity; the positive correlation remained highly significant when adjusted for age and T2DM duration. Interestingly, FGF21 appears to have similar mechanisms to lipid metabolism disorders in T2DM with HP. Correlation analysis showed that FGF21 was positively correlated with TG, negatively correlated with HDL-c, while no correlation with TC or LDL-c was found (Data not shown). Paradoxically, serum FGF21 in T2DM patients with HP was significantly higher than in control, which seems to contradict that the FGF21 can improve metabolic disorders. Many previous reports have confirmed that FGF21 is highly expressed in patients with chronic metabolic diseases such as obesity, diabetes, coronary heart disease, etc. The explanation of this paradox is that in these diseases, the human body has resistance to FGF21, and the sensitivity is reduced, which leads to the high compensatory increase of FGF21 in the circulation (34). FGF21 is primarily excreted by the kidney and is an independent risk factor for impaired kidney function. However, in the correlation analysis, FGF21 was not correlated with serum creatinine, blood urea nitrogen, serum uric acid, only negatively correlated with eGFR, after adjusting for age and sex, the correlation coefficients were reduced (Data not shown). This may be related to kidney function not being impaired in the early stage of diabetes. Moreover, it was reported that some kinds of anti-diabetic drugs have shown the potential effect the levels of FGF21. However, in the present study, no correlations were found between serum levels of FGF21 and anti-diabetic drugs use, possible confounding factors of anti-diabetic drugs that may hinder the obtained results were generally not considered.

Body shape parameters including weight, waistline, neck circumference, BMI, ABSI, WHR, and the proportion of abdominal obesity increased significantly. FGF21, waistline, age, HbA1c, and eGFR are independent risk factors for T2DM complicated with HP, suggesting that elderly individuals with abdominal obesity should pay more attention to weight control, not only blood-sugar control, to prevent the occurrence of HP, thus reduced the risk of cardiovascular death in patients with T2DM at high risk for cardiovascular events.

## 5. Limitations

This study has several limitations. First, the term “FGF21 resistance” during human obesity, in which circulating FGF21 levels are elevated, has been controversial partly due to difficulty in delineating the physiological and pharmacological mechanisms of FGF21 (10), weight loss and energy expenditure should be assessed to test chronic FGF21 sensitivity. However, FGF21 metabolism and signal transduction is a difficult challenge. Second, our present study is limited by the lack of measurements for coronary artery disease, in which FGF21 is significantly higher (35). Therefore, further studies are needed to elucidate the impact of coronary artery disease on the association of FGF21 and HP.

## 6. Conclusions

In this study, we have confirmed that the risk factors of diabetes complicated with HP are closely related to body shape, the frequency of chronic complications of diabetes such as HP is high, and an increased prevalence of abdominal obesity was observed with higher serum level of FGF21, in other words, FGF21 resistance occurs in obesity patients in T2DM with HP. The changes in FGF21 level in different populations showed the protective function of FGF21 on the circulatory system, such drugs targeting FGF21 for treating CVD and metabolic diseases will be an excellent boon for cardiovascular patients.

## Data Availability

The original contributions presented in the study are included in the article/Supplementary Material, further inquiries can be directed to the corresponding authors.
